# Clinical Significance of *CD200* and *CD56* Expression in Patients with Acute Myeloid Leukemia

**DOI:** 10.31557/APJCP.2020.21.3.743

**Published:** 2020-03

**Authors:** Salah Aref, Nashwa Abousamara, Emann El-Helaly, Mohamed Mabed

**Affiliations:** 1 *Department of Clinical Pathology, Hematology Unit, Faculty of Medicine, *; 2 *Hematology Unit, Oncology Center, Mansoura University, Egypt. *

**Keywords:** CD200, CD56, AML, Prognosis

## Abstract

**Background:**

Acute myeloid leukemia (AML) escape from immunosurveillance by immunosuppression. *CD200* and *CD56* expression represented an independent prognostic factor in many hematological malignancies but its importance in AML patients remains to be identified.

**Methods:**

*CD200* and *CD56* expression were assessed in the bone marrow blasts for Fifty-two (52) newly diagnosed AML by flowcytometry before start of therapy.

**Results:**

*CD200* ^+^ expression was reported in 28.8% of patients while 17.3% of patients showed *CD56*^+^ expression. M4 FAB revealed high frequency of both *CD200*^+^ and *CD56*^+^ expression. The overall survival of *CD200*^+^ patients was 19.2% compared to 35.3% in *CD200*^-^ (P= 0.049). On the other hand, *CD56*^+^ patients had the lowest complete remission rate (22.2% vs. 53.4%). In addition, *CD56*^+^ population had significant bad influence on overall survival than those of CD56^- ^population (11.1 % vs. 35.5 %, P= 0.047).

**Conclusions:**

*CD200* and *CD56* positive expression by myeloblasts at diagnosis denote poor prognostic indicator and correlated with poor cytogenetic findings. *CD200* could be used as therapeutic target in AML.

## Introduction

Acute myeloid leukemia (AML) is a clonal malignant disease of the hematopoietic tissue. The diversity of the clinical, the hematological and the genetic features among patients with AML has been recognized. Considerable progresses in defining new diagnostic and prognostic markers have been applied in AML treatment. The detection of specific molecules in the leukemic cells has special relevance and is mandatory for the identification of certain subtypes of myeloid neoplasms (Arber et al., 2016).

CD200 is a trans-membrane cell surface glycoprotein belonging to the type1 immunoglobulin super family (Wright et al., 2000). Expression of *CD200* is normally seen in some population of T and B-lymphocytes, neurons and endothelial cells (Wright et al., 2003). CD200 induces immunosuppression through engagement with CD200R, a cell-surface receptor homolog, which is expressed on leukocytes of myeloid lineage, including mast-cells, macrophages, basophils, dendritic cells as well as certain T-cell populations. CD200, which is frequently over expressed in AML blasts and is associated with a worse outcome. It has the potential to induce the formation of CD4^+^CD25^+^FoxP3^+^ regulatory T cells (Tregs), a subset of immunosuppressive T cells that are linked with a poor prognosis in AML (Coles et al., 2011).

Leukemic cells express leukemia-associated antigen, MHC, co stimulatory molecules and ligands for natural killer (NK) cells activating receptors, therefore leukemic cells are susceptible to be attacked by T and NK cells (el-Shami and Smith, 2008). CD56 antigen, a 200–220 kDa cell surface glycoprotein, identified as an isoform of the neural adhesion molecules (NCAM) (Gattenlöhner et al., 2009). CD56 firstly described as NK cell and then found in several hematopoietic malignancies including AML (Yoshida et al.,2015). CD56 was associated with poor prognosis in patients with acute myeloid leukemia (Alegretti et al., 2011). We herein, study the expression level of CD200 and CD56 in de-novo acute myeloid leukemia patients to estimate the prognostic value of their positive expressions individually in AML cases.

*Patients*

Between April 2014 and November 2015 pre-treatment bone marrow and peripheral blood, samples were obtained from 52 AML patients attending the oncology center, Mansoura University (OCMU). Their median age was 39.5 years (range 2 – 82 years). They were 29 male and 23 females. A written consent from all patients and an approval from the local institutional research committee were obtained. In order to confirm the presences of leukemic blast samples were screened morphologically and immunophenotypically

*Inclusion criteria*

Newly diagnosed AML patients. 

*Exclusion criteria*

Promyelocytic leukemia (M3)

Secondary acute myeloid leukemia

*Treatment*

Patients were treated according to the institutional approved protocols. Adult patients with AML, were treated with 3+7 protocol consists of 3 days doxorubicin (45mg/m^2^) and 7 days cytarabine (100-200 mg/m^2^ IV continuous infusion over 24 hours). Pediatric patients received five courses. The first two courses consist of Daunorubicin 50 mg/m^2^ IV, Cytosine arabinoside 100 mg/m^2^ IV, Etoposide 100 mg/m^2^ IV and Intrathecal cytarabine (age adjusted doses at time of diagnosis). The third course 3 MACE: Amsacrinea100 mg/m^2^ IV daily, Cytosine arabinoside 200 mg/m^2^/d IV, Etoposide 100 mg/m2 IV daily, and intra thecal cytarabine. The fourth course 4 MidAC: Mitoxantrone 10 mg/m^2^ IV daily and Cytosine arabinoside. The last Course 5 CLASP: Cytosine arabinoside 3.0 grams/m^2^ IV and L-asparaginase 6,000 IU/m^2^ IM. For patients with CNS disease at diagnosis IT therapy with cytarabine is given twice per week until CSF is clear with two additional doses after clearing of CSF with a minimum of 4 doses of intrathecal therapy.

## Materials and Methods

For AML diagnosis, broad panel of fluorochrome conjugated monoclonal antibodies (mAbs) were used that included: anti-CD3, CD4, CD5, CD7, CD8, CD10, CD13, CD14, CD19, CD20, CD22, CD33, CD34, CD117, HLADR, TDT. The gating was done using CD45 to determine the CD45 dim blast area. All monoclonal antibodies were purchased from BD Pharmingen, San Diego, CA, USA. 

Specific laboratory test for flow cytometric analysis of CD200 and CD56 were included for all patients using (PE CD200^- ^MRC OX-104) and (FITC CD56 NCAM16.2) antibodies [BD Pharmingen, San Diego, CA, USA]. They were used at concentrations recommended by their manufacturers. Samples were stained with monoclonal antibodies against cell surface markers using stain – lyses - wash then direct immunofluorescence technique. 100μl of BM sample were added to 10μl of MoAb and the cell suspensions were then incubated for 15 min at 4^o^C. Gating on myeloblasts was based on CD45 versus side scatter analysis. The expression of *CD200* on AML blasts could be detected after exclusion of lymphocytes from analysis based on low side scatter and high *CD45 *expression. *CD200* and* CD56* expression were evaluated as percentage and designated as AML blasts with CD200^+^, CD56^+^ with a cut off ≥ 20% of gated cells. HLA-DR and CD34 were positive with >10% of the cells expressed those markers. 

*Statistical analysis*

All statistical analyses were performed using the SPSS software package and Graph Pad Prism (Graph Pad Software, Inc; San Diego, CA). Data were statistically described in terms of mean with range and mean ± SD. Comparison of quantitative parametric variables between studied groups were assessed using One-way Analysis of Variance (ANOVA), Student’s t-test, Chi-square and correlation coefficient study. Overall survival (OS) was calculated from the date of first diagnosis to death from any cause. Whereas, remission duration was calculated from the time of achievement of complete remission (CR) to time of relapse or death in CR. A probability value (p value) less than 0.05 was considered statistically significant.

## Results

*Patient’s characteristics*

As seen in Table 1, the study included 52 de novo AML patients. They were 29 males and 23 females. Their age ranged from 2 to 82 years with a median of 39.5 years. According to FAB classification, 22 patients (42.3%) showed M4 subcategory. The positive expression was detected in fifteen patients (28.8%) for *CD200* and 9 patients (17.3%) for *CD56*. 

The clinical and laboratory data of CD200^+^ patients were compared to those of CD200^–^ patients and the data are shown in Table 2. CD200^+^ patients presented more with bleeding tendency; fever (P=0.056); high LDH levels (P=0.06) and correlated to cytogenetic findings. On the other hand, CD56^+^ had no significant influence in demographic and clinical data Table 3.

*Response to induction chemotherapy*

As shown in Table 4, 16 patients (43.2%) achieved CR in CD200^–^ subgroup compared to 9 patients (60%) in CD200^+^ subgroup (P>0.05). Fourteen patients (37.8%) in CD200^–^ subgroup showed induction failure compared to 4 patients (26.7%) in CD200^+^ subgroup (P>0.05). Seven patients (18.9%) died during induction in CD200^–^ subgroup with a total number of deaths of 22 patients (59.5%) compared to 2 patients (13.3%) in CD200^+^ patients (P>0.05).

As shown in table 5, 23 patients (53.4%) achieved CR in CD56^–^ subgroup compared to 2 patients (22.2%) in CD56^+^ subgroup (P=0.048). Thirteen patients (30.2%) in CD56^–^ subgroup showed induction failure compared to 4 patients (44.4%) in CD56^+^ subgroup (P>0.05). Seven patients (18.6%) died during induction in CD56^–^ subgroup with a total number of deaths of 27 patients (62.8%) compared to 3 patients (33.3%) in CD56^+^ patients (P>0.05).

*AML patient’s survival analysis*

**Figure 1:**
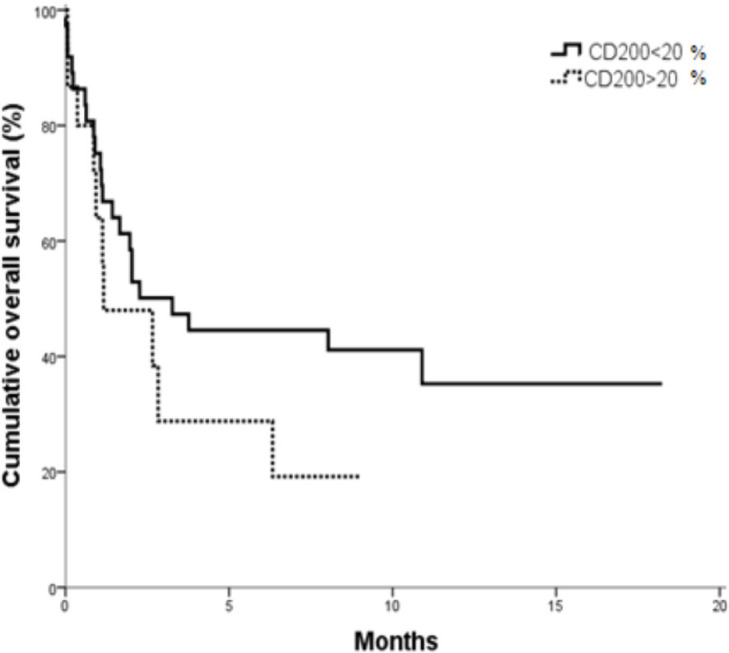
OS in CD200^+^ vs. CD200^- ^AML Patients (P=0.04).

The overall survival was significantly shorter in those having CD200^+^ compared to CD200^- ^ve, The cumulative overall survival after one year was 35.3% for CD200^– ^patients versus 19.2% for CD200^+^ patients. The Mean overall survival was 8.047 months (95 % CI; 5.379-10.715) for CD00^–^ compared to 3.224 months (95 % CI; 1.371-5.077) for CD200^+^ patients (P= 0.049) (Figure 1).

**Figure 2:**
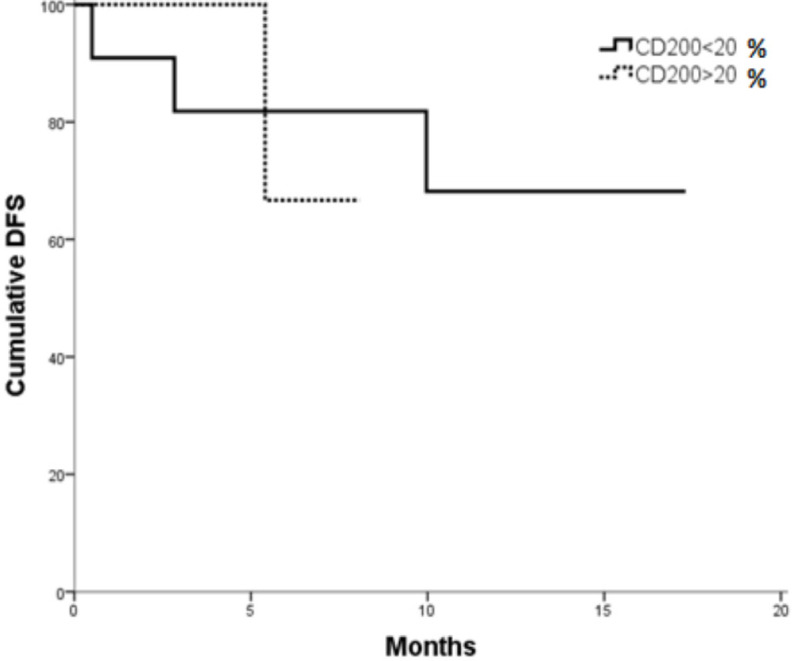
DFS in CD200^+^ vs. CD200^- ^Patients (p= 0.676)

Whereas, no significant differences were found in DFS according to CD200 expression in AML patients. The cumulative DFS after one year was 68.2% for CD200^–^ patients versus 66.7 % for CD200^+^ patients. The mean DFS was 13.457 months (95 % CI; 9.705-17.210) for CD00^–^ compared to 7.177 months (95 % CI; 5.755-8.599) for CD200^+^ patients (P= 0.676) (Figure 2).

**Figure 3:**
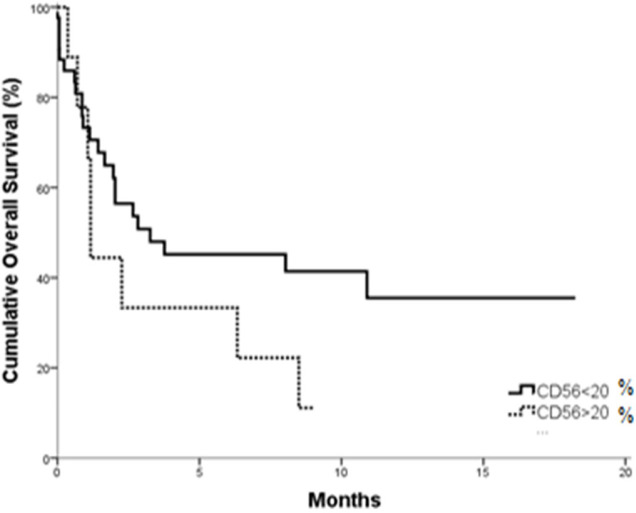
OS in CD56+ vs. CD56- AML Patients (P = 0.047).

AML patient with CD56^+^ showed significantly shorter OS. The overall survival was significantly shorter in those having CD56^+^ compared to CD56^-^, The cumulative overall survival after one year was 35.5% for CD56^–^ patients versus 11.1% for CD56^+^ patients. The mean overall survival was 8.168 months (95 % CI; 5.549-10.788) for CD56^–^ compared to 3.396 months (95 % CI; 1.227-5.564) for CD200^+^ patients (P= 0.047) (Figure 3).

**Figure 4:**
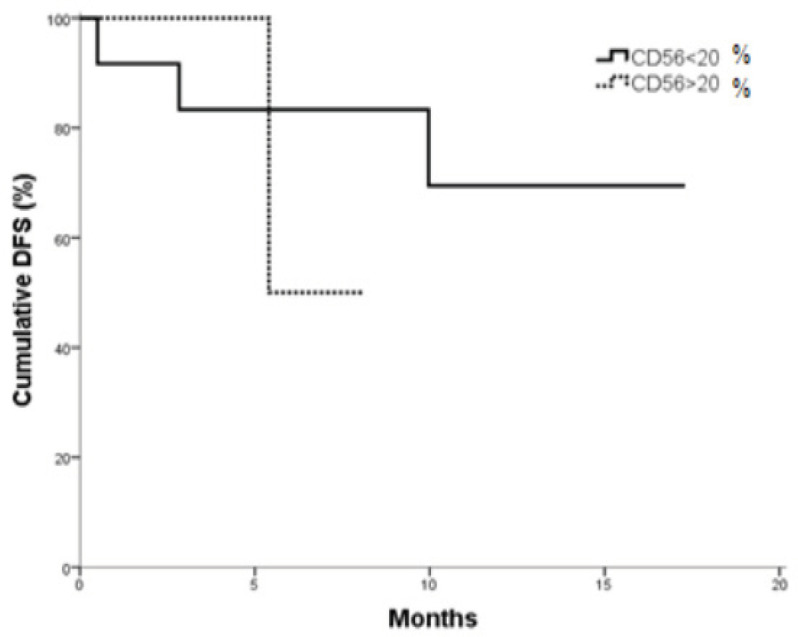
DFS in CD56+ vs. CD56- AML Patients (P = 0.047).

No significant differences were found in DFS according to CD56 expression in AML patients. The cumulative DFS after one year was 69.4% for CD 56^– ^patients versus 50% for CD56^+^ patients. The Mean DFS was 13.676 months (95 % CI; 0.122-17.229) for CD56^–^ compared to 6.733 months (95 % CI; 4.886-8.580) for CD56^+^ patients (P= 0.391) (Figure 4).

Cox regression analysis was conducted for prediction of survival times in all studied AML cases, using age, sex, BM blasts, LDH, CD200 and CD56 expressions as covariates. None of these covariates was associated with prediction of survival times in all studied AML cases [Data not shown].

**Table 1:** Patients’ Characteristics

Patients characteristics	No	%
Total number	52	
Age (years); median, range	39.5 (2-82)	
Gender:		
Males	29	55.8
Females	23	44.2
FAB classification:		
M0	1	1.9
M1	9	17.3
M2	12	23.1
M4	16	30.8
M5	9	15.5
M6	4	7.7
M7	1	1.9
CD200:		
+	15	28.8
-	37	71.2
CD56:		
+	9	17.3
-	43	82.7

**Table 2:** The Clinical and Laboratory Data in CD200^-^ AML Patients vs. CD200^+^ AML Patients

Parameter	CD200^-^ (N=37)	CD 200^+ ^(N=15)	P value
Median (range)			
Age (years)	39 (2-82)	49 (5-66)	0.8
Gender			
Males; N (%)	21 (56.8)	8 (53.3)	0.8
Females, N (%)	16 (43.2)	7 (46.7)	
Fever	28 (75.7%)	7 (46.7%)	0.056
Bleeding tendency	8 (21.6%)	9 (60.0%)	
WBCs (X10^9^/L)	27 (0.3-280)	63.9 (1.1- 188)	0.37
Hemoglobin level (g/dL)	7.5 (4.5 - 12.4)	6.9 (3.9-9.8)	0.26
Platelet count (X10^9^/L)	54 (9-344)	43 (12-101) 0.29	
Marrow blasts (%)	72 (23- 95)	85 (25-95)	0.23
LDH (U/L)	655.9 (155-2744)	1283.7 (658-5653)	0.06
Cytogenetic			
Favourable	22	2	0.01
Normal	15	4	
Unfavourable	0	9	

**Table 3:** The Clinical and Laboratory Data of CD56^-^ vs. CD56^+^ AML Patients

Parameter Median (range) CD56 – (n=43) CD56 ^+^ (n=9) P value
Age (years);39 (2-82) 40 (2-66) 0.75 >0.05
Gender
Males; N (%) 24 (55.8) 5 (55.6) >0.05
Females; N (%) 19 (44.2) 4 (44.4) >0.05
Fever 30 (69.8%) 5 (55.6%) >0.05
Bleeding manfestation 12 (27.9%) 5 (55.6%) >0.05
Total leucocytic count (X10^9^/L) 33.3 (1.1- 280.0) 17.1 (0.3- 188) >0.05
Hemoglobin concentration (g/dL) 7.5 (3.9 – 9.80) 6.9 (4.7- 10.8) >0.05
Platelet count (X10^9^/L) 39 (9- 344) 44 (12- 126) >0.05
Marrow blasts (%) 80 (25- 95) 80 (23- 95) >0.05
LDH (U/L) 12,00 (155- 5653) 684.34 (656 – 1430) >0.05
Cytogenetic Findings
Favorable 34 2 0.01
Normal 7 1
Poor 2 6

**Table 4:** Treatment Results According to *CD200* Expression

Parameter CD200^–^ (n=37) CD200^+ ^(n=15) P value
CR; N (%) 16 (43.2) 9 (60.0) >0.05
IF; N (%) 14 (37.8) 4 (26.7) >0.05
ID; N (%) 7 (18.9) 2 (13.3) >0.05
Total Deaths; N (%) 22 (59.5) 10 (66.7) >0.05

CR, complete remission; IF, induction failure; ID, induction death

**Table 5:** Treatment Results According to *CD56* Expression

Parameter CD200 ^–^ (n=37) CD200^+ ^(n=15) P value
CR; N (%) 16 (43.2) 9 (60.0) >0.05
IF; N (%) 14 (37.8) 4 (26.7) >0.05
ID; N (%) 7 (18.9) 2 (13.3) >0.05
Total Deaths; N (%) 22 (59.5) 10 (66.7) >0.05

CR, complete remission; IF, induction failure; ID, induction death

## Discussion

AML cells exert direct immunosuppressive effects on NK cells mediated by immunosuppressive ligands or soluble factors and induce regulatory T lymphocytes (T reg) that weaken NK-cell responses (Ustun et al., 2011). CD200 associated with IVIG is an important component of NK (*CD56*^+^) suppression. CD200-dependent suppression appears to be mediated by a non-NK population that acts on NK cells by direct contact rather than indirectly through release of immunosuppressive cytokines (Clark et al., 2008). We herein clarify the prognostic relevance of CD200 and CD56 in AML patients.

CD200 is a widely distributed membrane protein that gives inhibitory signals through its receptor (CD200R) on myeloid cells (Akkaya et al., 2016). The CD200: CD200R1 inhibitory signaling pathway has been implicated in playing a prominent role in limiting inflammation. CD200R1 signaling inhibits the expression of proinflammatory molecules including tumor necrosis factor, interferons, and inducible nitric oxide synthase in response to selected stimuli (Vaine et al., 2014). 

Furthermore, many studies have addressed the role of *CD56* expression in hematological malignancies. In fact, this antigen is an isoform of the neural cell adhesion molecules (NCAM), has been recorded in several myeloproliferative disorders and acute leukemia (Raspadori, et al., 2001). Also, CD56 identified as a predisposing factor for extramedullary manifestation and granulocytic sarcoma which is almost always followed by bone marrow relapse and should be treated with aggressive reinduction chemotherapy and local irradiation (Byrd and Weiss, 1994). 

In the present study, *CD200*^+^ was expressed in 15 of 52 patients (28.8%) and *CD56* was expressed in 9 of 52 patients (17.3%). Tiribelli et al., (2017) showed positive rate of CD200 antigen expression in 54 AML patients 57.4% (31/54). Also, Tonks et al., (2007) reported that CD200 was upregulated in 43% of patients diagnosed with AML. Damiani et al., (2015) in a larger study of 224 patients reported positive *CD200 *expression in 56% of patients. Our results showed lower rate of positivity of CD200 that may be attributed to inclusion of newly diagnosed patients and exclusion of secondary leukemia which show increased rate of expression of *CD200* as discussed by Damiani et al., (2015). 

CD200^+^ patients have significantly higher frequency of bleeding tendency. Tiribelli et al., (2017) and Zhu et al., (2016 ) showed that there is no significant differences in CD200 antigen expression regarding sex and age of patients. Our results showed that *CD200*^+^ highly expressed in M4 group. Tonks et al., (2007) reported that CD200 up regulated in almost all M2 and M4 FAB types.

AML patients with positive CD200 showed shorter overall survival as compared to AML patients with negative CD200 (P =0.04). Whereas, no significant differences were found in DFS and CR according to CD200 expression in AML patients. These results agreed with that reported by Tonks et al., (2007) and Damiani et al., (2015) as regarding to OS but the later showed that there was reduced probability to attain CR in CD200^+^ compared to CD200^- ^AML patients.

Overall survival in CD200^- ^group was significantly longer than that in CD200^+^ group. The expression of CD200^+^ positively correlates with the percentage of Treg in multiple myeloma patients (Zhu et al.,2016). Aref et al., (2015) illustrated that CD200^-^ MM patients had a better progression free survival and overall survival as compared with those positive for* CD200* expression. These findings also go with *CD200 *expression level and the frequency of immunosuppressive Treg cells.

CD200 affects patient’s immune response and a key immunosuppressive molecule. *CD200* expression on tumor cells inhibits the ability of human lymphocytes to eradicate tumor cells by interaction of CD200 with its receptor alters cytokine profiles from Th1 cytokines like (IFNS, IL2) to Th2 cytokines (IL10, IL4) in mixed lymphocyte reactions, and results in the induction of regulatory T cells, which are thought to slow down tumor-specific effector T cell immunity. This was confirmed by Kretz-Rommel et al., (2007). 

CD200 can suppress both the magnitude and intensity of the memory Th1response in AML. CD200 on AML cells directly impairs NK cell function. So, blocking CD200 alone was sufficient to recover a significant proportion of NK cell cytolytic activity accompanied with inferior overall survival and bad prognosis in AML patients with CD200^+^ (Wright et al., 2003). 

The expression of CD56 is considered a bad prognostic factor for overall survival, lower rates or short complete remission and extramedullary invasion in AML patients. Our results showed the expression rate of *CD56* in AML patients was 17.3%. This rate is similar to the previous result established by Alegretti et al.,(2011); who found 8 out of 48 patients expressed *CD56* (16.7%). In Ferrara et al. study; they detected 15% positively expressed *CD56*. While in study done by Raspadori et al., (2011); they reported that the positive expression of *CD56* was 37 out of 152 cases (24%).

Treatment results revealed that there was significant decrease in patients who achieved complete remission being low in CD56^+^ group vs. CD56^- ^group. This result achieved by Alegretti et al., (2011) in agreement with our results. In addition, Coelho-Silva et al., (2017) showed that there was significant difference regarding CR between CD56^+^ and CD56^- ^groups.

The AML overall survival is markedly shortened in CD56^+^ group. Whereas, DFS showed no significant difference between CD56^+^ and CD56^- ^groups. These results are in agreement with Raspadori et al., (2001). Whereas, evidences suggested that CD56 positive blasts may emerge from less-differentiated leukemic stem cells (Chang et al., 2004) and are less sensitive to standard chemotherapy schemes. Furthermore, co-expression with multidrug resistance (Raspadori et al., 2001) and extramedullary infiltrates (Coelho-Silva et al., 2017) are frequent findings in CD56^+^ patients and may underlie the adverse outcomes predicted by CD56 antigen. The limitation of this study is the small sample size of the AML patients.

In conclusion, *CD200*^+^ and *CD56*^+^ expression in AML at diagnosis are poor prognostic indicators and correlated with poor cytogenetic findings. CD200 could be used as therapeutic target in AML patients.

## Conflict of interest

The authors declare that there is no conflict of interest.
